# Supplementary material for: Effectiveness of a multifaceted prevention programme for melioidosis in diabetics (PREMEL): A stepped-wedge cluster-randomised controlled trial
Source: PLoS Negl Trop Dis. 2021 Jun 25;15(6):e0009060. doi: 10.1371/journal.pntd.0009060 (PMC8266097; doi:10.1371/journal.pntd.0009060)
Supplement: S1 Table — (DOCX) [file pntd.0009060.s001.docx]

**S1 Table. ICD-10-TM* codes and criteria used to determine that hospitals admissions were possibly related to infectious diseases.**

| ICD-10-TM | Diseases | n ** |
| --- | --- | --- |
| A00-B99 | Certain infectious and parasitic diseases*** | 1213 (36%) |
| D73.3 | Abscess of spleen | 20 (0.6%) |
| H10, H16, H44.0 | Eye infection | 14 (0.4%) |
| H60, H65, H66 | Infective otitis externa | 17 (1%) |
| I01, I33, I38, I39 | Endocarditis | 9 (0.3%) |
| J00-J06 | Acute upper respiratory infections | 88 (3%) |
| J09-J18 | Influenza and pneumonia**** | 372 (11%) |
| J20-J22 | Other acute lower respiratory infections | 51 (2%) |
| J85-J86 | Suppurative and necrotic conditions of the lower respiratory tract | 11 (0.3%) |
| K75.0 | Abscess of liver | 11 (0.3%) |
| K80, K81 | (Acute) cholecystitis | 104 (3%) |
| L00-L08, L30.3, L66.3, L66.4 | Infections of the skin and subcutaneous tissue***** | 452 (14%) |
| M00-M02, M86 | Bone and join infections | 91 (3%) |
| N13.6, N15.1,  N30, N39.0 | Urinary tract infection | 287 (9%) |
| R50.9 | Fever, unspecified | 241 (7%) |
| R57.2 | Septic shock | 143 (4%) |
| T79.3 | Post-traumatic wound infection, not elsewhere classified | 302 (9%) |
| T85.7 | Infection and inflammatory reaction due to other internal prosthetic devices, implants and grafts | 50 (1%) |
| Others | Having terms “fever, febrile, infect, abscess, pus, diarrhea, sepsis, pneumonia, cellulitis or diabetic foot” in a primary diagnosis | 1882 (56%) |
| Any ICD-10 codes | Culture positive for *Burkholderia pseudomallei* from any clinical specimens | 57 (2%) |
| Total | - | 3,335 |

* ICD-10-TM = International Statistical Classification of Diseases and Related Health Problems, 10th Revision, Thailand Modification ** Data are n (%) of 3,335 admissions involving infectious diseases. Codes were de-duplicated per hospital admission. *** Included A09 (diarrhoea and gastroenteritis of presumed infectious origin; n=582), A41 (sepsis, n=179), A24 (melioidosis, n=76), A05.9 (Bacterial foodborne intoxication, n=61), A49.9 (Bacterial infection, unspecified, n=42), etc. **** Included J18 (pneumonia, unspecified organism, n=264), J15 (bacterial pneumonia, not elsewhere classified, n=103), J209 (acute bronchitis, n=40), etc. ***** Included L03 (Cellulitis, n=251), L024 (cutaneous abscess, n=76) and L089 (Local infection of the skin and subcutaneous tissue, unspecified, n=52), etc.
