# Supplementary material for: Effectiveness of a multifaceted prevention programme for melioidosis in diabetics (PREMEL): A stepped-wedge cluster-randomised controlled trial
Source: PLoS Negl Trop Dis. 2021 Jun 25;15(6):e0009060. doi: 10.1371/journal.pntd.0009060 (PMC8266097; doi:10.1371/journal.pntd.0009060)
Supplement: S3 Table — (DOCX) [file pntd.0009060.s003.docx]

**S3 Table. Factors associated with hospital admissions involving infectious diseases**

| **Factors** | **Adjusted rate ratio (95% CI)*** | **P value** |
| --- | --- | --- |
| Received the intervention per protocol | 0.90 (0.81-1.00) | 0.04 |
| Time period |  |  |
| Period 1 (Apr 2014 – Feb 2015) | 1.00 | <0.001 |
| Period 2 (Mar 2015 – Feb 2016) | 1.21 (1.05-1.39) |  |
| Period 3 (Mar 2016 – Feb 2017) | 1.51 (1.31-1.73) |  |
| Period 4 (Mar 2017 – Feb 2018) | 1.95 (1.69-2.25) |  |
| Period 5 (Mar 2018 – Dec 2018) | 2.13 (1.83-2.49) |  |
| Sex, female | 0.72 (0.64-0.80) | <0.001 |
| Age |  |  |
| 18 - <40 years | 1.00 | <0.001 |
| 40 - <55 years | 1.15 (1.02-1.31) |  |
| 55 – 65 years | 1.42 (1.24-1.63) |  |
| Diabetes duration |  |  |
| <5 years | 1.00 | <0.001 |
| 5 - <10 years | 1.22 (1.07-1.38) |  |
| ≥10 years | 1.99 (1.76-2.24) |  |
| HbA_1c_ level |  |  |
| <7.0 % | 1.00 | <0.001 |
| 7.0 - 8.0% | 1.06 (0.92-1.23) |  |
| >8.0 - 9.0% | 1.20 (1.02-1.41) |  |
| >9.0% | 2.27 (1.97-2.62) |  |

* CI=confidence interval. Estimated using a multivariable multilevel mixed-effect negative binomial regression model with a random effect for PCU (n=9,056 diabetic patients)
