# Supplementary material for: Effectiveness of a multifaceted prevention programme for melioidosis in diabetics (PREMEL): A stepped-wedge cluster-randomised controlled trial
Source: PLoS Negl Trop Dis. 2021 Jun 25;15(6):e0009060. doi: 10.1371/journal.pntd.0009060 (PMC8266097; doi:10.1371/journal.pntd.0009060)
Supplement: S4 Table — (DOCX) [file pntd.0009060.s004.docx]

**S4 Table. Factors associated with mortality**

| **Factors** | **Adjusted rate ratio (95% CI)** | **P value** |
| --- | --- | --- |
| Received the intervention per protocol | 0.56 (0.44-0.71) | <0.001 |
| Time period |  |  |
| Period 1 (Apr 2014 – Feb 2015) | 1.00 | <0.001 |
| Period 2 (Mar 2015 – Feb 2016) | 1.39 (0.93-2.08) |  |
| Period 3 (Mar 2016 – Feb 2017) | 2.07 (1.40-3.07) |  |
| Period 4 (Mar 2017 – Feb 2018) | 3.01 (2.04-4.44) |  |
| Period 5 (Mar 2018 – Dec 2018) | 3.42 (2.27-5.15) |  |
| Sex, female | 0.42 (0.35-0.51) | <0.001 |
| Age |  |  |
| 18 - <40 years | 1.00 | <0.001 |
| 40 - <55 years | 1.40 (1.08-1.81) |  |
| 55 – 65 years | 2.09 (1.60-2.72) |  |
| Diabetes duration |  |  |
| <5 years | 1.00 | <0.001 |
| 5 - <10 years | 1.30 (1.02-1.66) |  |
| ≥10 years | 2.03 (1.62-2.56) |  |
| HbA_1c_ level |  |  |
| <7.0 % | 1.00 | <0.001 |
| 7.0 - 8.0% | 0.82 (0.62-1.08) |  |
| >8.0 - 9.0% | 1.16 (0.87-1.54) |  |
| >9.0% | 1.40 (1.09-1.80) |  |

* CI=confidence interval. Estimated using a multivariable multilevel mixed-effect Poisson regression model with a random effect for PCU (n=9,056 diabetic patients)
