# Supplementary material for: Effectiveness of a multifaceted prevention programme for melioidosis in diabetics (PREMEL): A stepped-wedge cluster-randomised controlled trial
Source: PLoS Negl Trop Dis. 2021 Jun 25;15(6):e0009060. doi: 10.1371/journal.pntd.0009060 (PMC8266097; doi:10.1371/journal.pntd.0009060)
Supplement: S5 Table — (DOCX) [file pntd.0009060.s005.docx]

**S5 Table. Factors associated with overall melioidosis**

| **Factors** | **Adjusted rate ratio (95% CI)*** | **P value** |
| --- | --- | --- |
| Received the intervention per protocol | 0.74 (0.38-1.44) | 0.37 |
| Time period |  |  |
| Period 1 (Apr 2014 – Feb 2015) | 1.00 | 0.41 |
| Period 2 (Mar 2015 – Feb 2016) | 0.73 (0.34-1.54) |  |
| Period 3 (Mar 2016 – Feb 2017) | 1.34 (0.65-2.73) |  |
| Period 4 (Mar 2017 – Feb 2018) | 0.87 (0.38-2.08) |  |
| Period 5 (Mar 2018 – Dec 2018) | 0.95 (0.34-2.67) |  |
| Sex, female | 0.28 (0.18-0.44) | <0.001 |
| Age |  |  |
| 18 - <40 years | 1.00 | 0.12 |
| 40 - <55 years | 0.74 (0.44-1.22) |  |
| 55 – 65 years | 0.52 (0.28-0.98) |  |
| Diabetes duration |  |  |
| <5 years | 1.00 | 0.005 |
| 5 - <10 years | 1.21 (0.66-2.21) |  |
| ≥10 years | 2.37 (1.36-4.12) |  |
| HbA_1c_ level |  |  |
| <7.0 % | 1.00 | <0.001 |
| 7.0 - 8.0% | 1.71 (0.77-3.79) |  |
| >8.0 - 9.0% | 0.90 (0.33-2.45) |  |
| >9.0% | 3.42 (1.64-7.15) |  |

* CI=confidence interval. Estimated using a multivariable multilevel mixed-effect Poisson regression model with a random effect for PCU (n=9,056 diabetic patients)
