# Supplementary material for: Effectiveness of a multifaceted prevention programme for melioidosis in diabetics (PREMEL): A stepped-wedge cluster-randomised controlled trial
Source: PLoS Negl Trop Dis. 2021 Jun 25;15(6):e0009060. doi: 10.1371/journal.pntd.0009060 (PMC8266097; doi:10.1371/journal.pntd.0009060)
Supplement: S6 Table — (DOCX) [file pntd.0009060.s006.docx]

**S6 Table. Outcomes of the study excluding infections that are not plausibly related to the intervention***

|  | Adjusted (for time) incidence rate ratio (95% CI) | Adjusted (for time and other risk factors**) incidence rate ratio (95% CI) |
| --- | --- | --- |
| **Intention-to-treat analysis** |  |  |
| Hospital admissions involving infectious diseases | 0.96 (0.84-1.10) | 0.96 (0.83-1.09) |
| **Per-protocol analysis ***** |  |  |
| Hospital admissions involving infectious diseases | 0.88 (0.78-0.99) | 0.89 (0.79-1.00) |

CI=confidence interval

* A pre-specified analysis excluding certain infectious and parasitic diseases (A15-A23, A25-A48, A50-A99, B00-B64, B85-B97), eye infection (ICD-10-TM H10, H16, H44.0), Infective otitis externa (ICD-10-TM H60, H65, H66), endocarditis (ICD-10-TM I01, I33, I38, I39), acute upper respiratory infections (ICD-10-TM J00-J06), influenza, viral pneumonia, pneumonia due to *Streptococcus pneumoniae*, *Haemophilus influenzae* and other specified infectious organisms (ICD-10-TM J09-J14, J16), acute bronchitis and acute bronchiolitis (ICD-10-TM J20-J21), (acute) cholecystitis (ICD-10-TM K80, K81), urinary tract infection (ICD-10-TM N13.6, N15.1, N30, N39.0) and infection and inflammatory reaction due to other internal prosthetic devices, implants and grafts (ICD-10-TM T85.7)

** Adjusted for age, sex, known diabetes duration and HbA1c level

*** Participants who received a behavioural support group session for melioidosis prevention were defined as received the intervention per protocol.
