# Supplementary material for: Effectiveness of a multifaceted prevention programme for melioidosis in diabetics (PREMEL): A stepped-wedge cluster-randomised controlled trial
Source: PLoS Negl Trop Dis. 2021 Jun 25;15(6):e0009060. doi: 10.1371/journal.pntd.0009060 (PMC8266097; doi:10.1371/journal.pntd.0009060)
Supplement: Attachment — Submitted filename: response BCT 2021 04 03.docx [file pntd.0009060.s007.docx]

**Re: Decision on your PLOS Neglected Tropical Diseases submission (PNTD-D-20-02241) - [EMID:d9180f9708603eef]**

Dear Editor,

Thank you for your e-mail concerning the above manuscript. We are grateful for the helpful comments made by the reviewers, and have addressed them as detailed below.

Editor’s comments:

The reviewers recommend substantial changes to the manuscript, including the conclusion, recommending caution in not over-stating the potential benefits of the intervention, and justification for inclusion of melioidosis cases that were not microbiologically confirmed. Despite the negative findings for the study, these are potentially important findings to share after addressing the reviewers’ comments. The authors could discuss why the interventions did not result in significant differences, with respect to study design or biological factors.

***Answer:* The conclusion has been revised to “Clear benefits of the multifaceted prevention programme for melioidosis could not be observed. Stronger invitations for the intervention, modification or addition of the behaviour change techniques used, and more frequent intervention may be needed. Further studies are required.” The discussion has been revised as suggested.**

Reviewers’ comments:

-Are the objectives of the study clearly articulated with a clear testable hypothesis stated? -Is the study design appropriate to address the stated objectives? -Is the population clearly described and appropriate for the hypothesis being tested? -Is the sample size sufficient to ensure adequate power to address the hypothesis being tested? -Were correct statistical analysis used to support conclusions? -Are there concerns about ethical or regulatory requirements being met?

Reviewer #1: I am satisfied with the methods that have been employed.

The statistical analysis is very complicated and I lack the expertise to offer a sensible comment on the statistical methods that have been employed. I hope that one of the other reviewers have more expertise so that this might be critically assessed.

Reviewer #2: All good

***Answer:*** **We are grateful for the comments.**

--------------------

-Does the analysis presented match the analysis plan? -Are the results clearly and completely presented? -Are the figures (Tables, Images) of sufficient quality for clarity?

Reviewer #1: The results are presented in a detailed manner and in general, I am happy with them.

Although I might suggest that the authors try not to duplicate results in the text and the tables/figures. For instance, lines 278-281 is repeated - almost word for word - in figure 1 and doesn't add much to the study's interpretation (0.2% of the cohort were excluded). There are several other examples where data are duplicated in text and in a table or figure like this.

Reviewer #2: Yes

***Answer:* Lines 278-281 have been removed. Additionally, lines 295-297 have been removed to avoid repetition between texts and tables as suggested.**

--------------------

-Are the conclusions supported by the data presented? -Are the limitations of analysis clearly described? -Do the authors discuss how these data can be helpful to advance our understanding of the topic under study? -Is public health relevance addressed?

Reviewer #1: This is my major concern with the paper.

I think the authors have performed an impressive study, but they have over-egged their - in my opinion - negative results. To me the studied intervention - if significant at all - appears to be a very low-value intervention.

The primary aim of the study was to evaluate the effectiveness of a prevention programme for melioidosis.

22/80 (27.5%) of the patients who were said to have melioidosis had a "clinical diagnosis".

As the authors themselves note in lines 94-95, a positive culture for B. pseudomallei is the gold standard for diagnosis.

So why then do they include the "clinical diagnosis" of melioidosis cases in their analysis? I would be quite confident that a significant proportion of the patients with "clinical melioidosis" did not actually have melioidosis. How then to explain that their case-fatality rate was 0 (compared to 26% among the culture-confirmed group). Even in the very well-resourced Australian health system the case-fatality rate of melioidosis is about 10% of those admitted. I would suggest that the reason that the case-fatality rate in the patients with "clinical melioidosis" is so low is that many did not actually have melioidosis. Indeed, the authors state that the reason that you would want to prevent melioidosis is that it is often fatal; in lines 86-88 they cite a modelling paper that suggests that 54% of cases of melioidosis die.

The patients with "clinical melioidosis" in this cohort actually did pretty well! If this is the case, perhaps there is less need to worry about preventing it?

Even including the clinically diagnosed cases in a per-protocol analysis in a sample of 9075 patients the IRR is 0.57 (95%CI 0.31-1.08), p=0.09. This is not, as the authors state, "borderline evidence of a lower incidence rate" it is statistically not significant. Even if the authors suggest that this "trend" is a type 2 error, the size of the sample that you would need to prove that the effect was statistically significant suggests that the actual impact of the intervention is quite limited. If you can’t show it in 38,457 person-years of follow-up, any putative benefit much be tiny. What is the number needed to treat (NNT)?

The two main interventions that the authors were keen to implement (drinking bottled/boiled water and wearing boots - lines 172-173) were said to reduce hospital admissions for infectious diseases (IRR 0.89 (95%CI 0.80-0.99, p=0.03), but how much was this simply determined by gastroenteritis admissions, (the most common cause for admission with infection) which could just be explained by getting the participants to drink boiled/bottled water. No need to fuss around with all the education about wearing boots. Additionally, how do the authors propose the wearing of boots and drinking bottled/boiled water reduce urinary tract infections?

The patients who did not receive the intervention were more likely to be male and more likely to have poorer glycaemic control (among other significant differences). Although the authors have used complicated statistical methods to control for the contribution of these factors, lacking the statistical knowledge to critically appraise this, I would want to be confident that the patients who declined to receive or wait around for the intervention are not just the patients who are going to make poorer health decisions anyway. Male gender is a recognised risk factor for poor health outcomes and poor glycaemic control may also be a proxy maker for poor access to care/adherence. Indeed, the authors do acknowledge this as a potential limitation in the study (lines 400-402). I think this discussion could be expanded.

***Answer:* We are grateful for the comments. The conclusion has been revised to “Clear benefits of the multifaceted prevention programme for melioidosis could not be observed. Stronger invitations for the intervention, modification or addition of the behaviour change techniques used, and more frequent intervention may be needed. Further studies are required.” Additional sensitivity analysis has been added. The discussion has been revised.**

Finally, I am not convinced by their health economic argument. The statistically non-significant benefit of the intervention would – the authors “estimate” – be cost-effective if policy makers were willing to pay $7000 per QALY. If this funding were directed elsewhere – to measures that improve glycaemic control for instance (lifestyle and pharmacological interventions) or prevent the development of diabetes, not only would this reduce the incidence of melioidosis but also the cardiovascular deaths and other complications of metabolic syndrome which have far more impact at a community level than melioidosis (56 confirmed cases in 38,457 years of patient follow up)

**Answer: The health economics part has been removed.**

Reviewer #2: Yes

--------------------

Summary and General Comments

Reviewer #1: I would like to congratulate the authors on an ambitious and impressive piece of work.

However, I am not at all convinced by the article's conclusions as they presently stand.

Even with 38,457 person-years of follow-up, using a per-protocol analysis and included "clinical" cases of melioidosis they have not been able to show that the intervention significantly reduced the incidence of melioidosis.

There was a reduction in infections requiring hospitalisation, but what proportion of the reduction in infectious diseases admissions can be explained by the bottled water intervention alone on the most common infection, gastroenteritis?

Improving access to clean water may be a simpler and cost-effective intervention at a population level.

The paper certainly merits publication and the melioidosis research community will find the work very interesting. However, I think revision of their conclusions, downplaying the putative success of the intervention significantly would make the paper more credible.

**Answer: The conclusion and discussion have been revised as suggested.**

Reviewer #2: The authors conducted a cluster randomised trial using step wedge design to study the impact of a behavioural intervention on hospitalisation due to infectious diseases and incidence of melioidosis among diabetic patients in Northern Thailand.

The authors acknowledged the imbalance in gender, and larger group size than predefined that may have influenced learning.

A few comments:

(1) Can the authors explain why recruitment ended in 2014 but randomisation only occurred in 2016?

**Answer: This was because we enrolled patients from April to November 2014 as stated in the results section. Therefore, we allowed year 2015 to be the control period, and started the intervention in March 2016.**

(2) Did the authors exclude all diabetic patients with known melioidosis? Exclusion criteria stated that those yet to complete antibiotic were excluded. If they did not, what proportion with known previous melioidosis was enrolled? Even with completion of coytrimoxazole, there is still a 5% relapse rate, which may have affected the incidence of melioidosis.

**Answer: No, we excluded only known melioidosis patients who had not yet completed oral eradicative treatment therapy. Additional results have been added as follows, “Fifty-three patients had history of culture-confirmed melioidosis and completed oral eradicative treatment prior to the enrollment.” and “Of 53 patients who had history of culture-confirmed melioidosis and completed oral eradicative treatment prior to the enrollment, none had culture-confirmed melioidosis and three had clinical melioidosis during the study period.”**

(3) Can the authors clarify why they did not conduct the intervention year round which could lead to better recruitment and better power as even if rainy season has started, behaviour change could still reduce infections? In fact, it may be more effective as the potential outcomes may be more real.

**Answer: We conducted the intervention during the dry season prior to the rainy season because we want to deliver the intervention before the start of the rainy season and before patients started working in the rice field. During the rainy season, many diabetic patients also tend to ask for two to three months of diabetic drugs so that they can come to hospital as little as possible due to their work in the rice fields.**

(4) One of the major weakness is the lack of monitoring and reminder with only a yearly phone call. Lost opportunity to reinforce at regular clinic visits for diabetes which are more frequent than yearly may have improved the outcomes. Can the authors explain why they decided only on yearly phone call? Why did they not ask for diary; reminder text messages; more frequent phone calls e.g. 3 monthly; reminder during rainy season?

**Answer: This was due to the limitation of human resources, time and budget. The study team had 8 research assistants, and the implementation of the intervention, conducting yearly phone calls, and following data on hospital admissions of 9,000 participants already required the full capacity of the study team. We also provided calendars as a reminder so that they could note their activities on a daily basis. Additional discussions about limitations have been added as follows, “One of the major weakness is the lack of monitoring and reminder with only a yearly phone call. Lost opportunity to reinforce at regular clinic visits for diabetes which are more frequent than yearly may have improved the outcomes. Had we had more human resources, time and budget, we would have included more phone calls and reminder text messages including during the rainy season. The study team had eight research assistants, and the implementation of the intervention, conducting yearly phone calls, and following data on hospital admissions of 9,000 participants already required the full capacity of the study team. We also provided calendars as a reminder so that they could note their activities on a daily basis.”**

(5) Can the authors explain why they thought obtaining cause of death from relatives may contribute to reliable data on secondary outcome of mortality? What was the average educational background of the family members?

**Answer: By obtaining cause of death from relatives, we could exclude some obvious non-infectious causes such as motorcycle accidents and we could obtain what information the relatives were given by doctors during the last visits prior to death. The educational background of the family members was roughly equivalent to US grade 6.**

(6) Primary outcome included infections like urinary tract infections that are not plausibly related to wearing boots and drinking boiled or bottled water. These are also not in pre-defined primary outcome. Can the authors explain the basis of inclusion? Can they do sensitivity analysis by excluding infections that are not plausibly related?

**Answer: We conducted the analysis based on the pre-determined protocol as published at clinicaltrials.gov. We have conducted a sensitivity analysis as suggested, and additional results have been added as follows, “We also conducted an analysis excluding hospital admissions involving infections that are not plausibly related to the intervention such as urinary tract infections, and similar results were observed (Supplementary Table 6).”**

(7) There is no effect on melioidosis. I think the authors should accept that and not claim borderline or trend to siginficance in any part of the manuscript including abstract.

**Answer: The conclusion and discussion have been revised as suggested.**

***All contributing authors have reviewed and concurred with the revised manuscript.***

***Yours,***

***Direk Limmathurotsakul***
