# Supplementary material for: Effectiveness of a multifaceted prevention programme for melioidosis in diabetics (PREMEL): A stepped-wedge cluster-randomised controlled trial
Source: PLoS Negl Trop Dis. 2021 Jun 25;15(6):e0009060. doi: 10.1371/journal.pntd.0009060 (PMC8266097; doi:10.1371/journal.pntd.0009060)
Supplement: Attachment — Submitted filename: response BCT 2021 05 03_ND.docx [file pntd.0009060.s008.docx]

**Re: Decision on your PLOS Neglected Tropical Diseases submission (PNTD-D-20-02241R1) - [EMID:47560d3c0cd1386f]**

Dear Editor,

Thank you for your e-mail concerning the above manuscript. We are grateful for the helpful comments made by the reviewers, and have addressed them as detailed below.

Editor’s comments:

Dear authors, thank you for your major revision that has addressed the major issues pointed out by both reviewers. To further improve the clarity and message of the manuscript, please address the 3 points raised by reviewer 1, and if there is disagreement with the reviewer, please justify. The public message from this study remains clear: that successful, low-cost interventions are challenging in this community and the authors should spare a few more sentences to describe the socioeconomic challenges and perhaps new avenues of intervention, eg through better control of diabetes or targeted use of antimicrobial prophylaxis (e.g. see https://pubmed.ncbi.nlm.nih.gov/29340327/) or water treatment (e.g. https://www.ncbi.nlm.nih.gov/pmc/articles/PMC3741262/), that may be more effective.

**Answer: The conclusion has been revised as suggested; “In conclusion, clear benefits of this multifaceted prevention programme for melioidosis were not observed. Successful and cost-effective interventions are still needed though challenging to design and implement. We propose that more compelling invitations for the intervention, modification of or addition to the behaviour change techniques used, and more frequent intervention may be needed. In addition, alternative interventions including targeted use of antimicrobial prophylaxis [32], and more general clinical and public health interventions such as better control of diabetes and improvement of water treatment [33] should also be considered.”**

Another minor point: Please delete the last sentence "Further study may be required" from the abstract as it adds no value.

**Answer: The abstract has been revised as suggested.**

Reviewers’ comments:

Reviewer #1: Thank you for the opportunity to review the revised manuscript by Limmathurotsakul et al. I appreciate the time that they have take to address the reviewers’ comments. I have only a few comments to make.

While they have pleasingly re-presented the study as a negative one, rather than a borderline positive one, I would suggest that they have not gone quite far enough, but I will leave it to the Editor to adjudicate!

1. In the abstract they say that in the per-protocol analysis the patients receiving the intervention had a lower incidence of hospital admissions involving infectious diseases (OR: 0.89, 95%CI 0.80-0.99, p=0.03).

In supplementary table 6, they present an OR of 0.89, (95%CI 0.80-1.0) and do not present a p value (that I can easily see).

They also say that they have “Excluded certain infectious and parasitic diseases (A15-A23, A25-A48, A50-A99, B00-B64, B85-B97), eye infection (ICD-10-TM H10, H16, H44.0), Infective otitis externa (ICD-10-TM H60, H65, H66), endocarditis (ICD-10-TM I01, I33, I38, I39), acute upper respiratory infections (ICD-10-TM J00-J06), influenza, viral pneumonia, pneumonia due to Streptococcus pneumoniae, Haemophilus influenzae and other specified infectious organisms (ICD-10-TM J09-J14, J16), acute bronchitis and acute bronchiolitis (ICD-10-TM J20-J21), (acute) cholecystitis (ICD-10-TM K80, K81), urinary tract infection (ICD-10-TM N13.6, N15.1, N30, N39.0) and infection and inflammatory reaction due to other internal prosthetic devices, implants and grafts (ICD-10-TM T85.7)” that are not plausibly related to the intervention (boots and bottled water)

However, I think I am right to say that they do not seem to have excluded a variety of infections that it would be quite a stretch to say are prevented by boots and bottled water. These would appear to include bone and joint infections and fever “unspecified”, among others.

It appears that they are just keen to show “some” effect of the intervention.

My strong advice would be to delete this from the abstract; if they are determined to link their intervention to a reduction in infectious diseases admissions, please list the infections that they are saying HAVE been prevented by the boots and bottled water (which I would argue might be limited to skin and soft tissue infections of the feet and their complications and gastroenteritis). Then present the odds ratio for the reduction in the incidence of these diseases.

**Answer: We would like to keep the results presented in the abstract and main text as they were based on the proposed analysis plan as registered with ClinicalTrials.gov, number NCT02089152, prior to enrollment of the first participant. We consider that any additional ad-hoc sensitivity analyses would not be robust and should be avoided. We are thankful for the reviewer’s concern. Additional sentence has been added in the limitation for clarity, “ICD coding can also be diagnostically inaccurate because of the subjective nature of ICD certification practices and limited training. The association between receiving a behavioural support group session for melioidosis prevention and lower rates of hospital admissions related to infectious diseases was not strong, and the impact of the intervention on hospital admission related to infectious diseases should be considered carefully.”**

2. I really think they should delete all references to “clinical melioidosis” in the paper. I am cognisant of the challenges of obtaining a microbiological diagnosis in regional Thailand, but to the melioidosis audience (who will be the main readers of the paper), this just reduces the paper's credibility. Especially as the case-fatality rate in these 25 patients was 0%. Even in Australia’s well resourced health system with access to ICU and ECMO the case fatality rate of hospitalised patients is about 10%. This suggests that many - if not most - of these patients with “clinical melioidosis” don’t have melioidosis at all. The CFR in the confirmed cases in this cohort was 26%, which is similar to relevant studies from the literature.

Alternatively, the authors could produce a very interesting case series on how they managed to reduce the CFR to 0% in these clinical melioidosis patients. We’d be interested to know!

**Answer: We would like to keep the results presented in the abstract and main text as they were based on the proposed study analysis plan as registered with ClinicalTrials.gov, number NCT02089152, prior to enrollment of the first participant. The low mortality of those clinical melioidosis cases could be a spurious finding (because the sample size of clinical melioidosis was small (n=22)), early management (because they were study participants) and, as the reviewer says, inaccurate final diagnosis in a proportion of those patients.**

3. The authors have had a really good go at providing an intervention that will reduce the incidence of melioidosis. They are to be commended for their efforts and the work is a valuable addition to the melioidosis literature.

However, fundamentally this is a negative study. If they cannot show a benefit of the intervention in the controlled study environment, how would prevention strategies fare in the Real-World setting?

About 30% of the patients in the cohort had poor glycaemic control, so they are struggling with their diabetes management. Meanwhile, 27% of enrolled patients did not have the time/understanding/interest to attend the education session.

Delivering interventions to this population is obviously going to be challenging, yet this does not seem to have been acknowledged, rather “Stronger invitations for the intervention, modification or addition of the behaviour change techniques used, and more frequent intervention may be needed”.

I do not want to seem nihilistic, but I think given that despite the dedicated work of the study team, they could not show a benefit. Therefore I would think at least a sentence or two about the cost-effectiveness of melioidosis prevention at a population level would be appropriate. My back of the envelope calculations suggest that culture-confirmed melioidosis occurred in this cohort at a rate of 151/100,000/year. Would finite health Dollars/Baht be better spent on other public health strategies that address the social determinants of health, health literacy and access to care that affect not only melioidosis but other communicable and non-communicable diseases?

**Answer: The conclusion has been revised as follows; “In conclusion, clear benefits of this multifaceted prevention programme for melioidosis were not observed. Successful and cost-effective interventions are still needed though challenging to design and implement. We propose that more compelling invitations for the intervention, modification of or addition to the behaviour change techniques used, and more frequent intervention may be needed. In addition, alternative interventions including targeted use of antimicrobial prophylaxis [32], and more general clinical and public health interventions such as better control of diabetes and improvement of water treatment [33] should also be considered.”**

***All contributing authors have reviewed and concurred with the revised manuscript.***

***Yours,***

***Direk Limmathurotsakul***
